# Principles of Human Physiology, with Their Chief Applications to Pathology, Hygiene and Forensic Medicine

**Published:** 1850-01

**Authors:** 


*Principles of Human Physiology, with their chief applications to Pathology, Hygiene and Forensic Medicine.* By William B. Carpenter, M. D., F. R. S., F. G. S. &c., &c. *Fourth American Edition, with extensive additions and improvements by the Author, with two plates and three hundred and four woodcuts.* Philadelphia, Lea & Blanchard, 1850.

Were any proof of the popularity of Dr. Carpenter's work in this country required, the fact of there being a call for a fourth American, before the third English Edition was exhausted would be sufficient. Since 1842 four large editions have been printed, three of which have met with a rapid sale. In the present instance, as was mentioned above, the demand for a new issue in the United States, has anticipated the exhaustion of the English Edition. Instead of merely reprinting the last Edition, or entrusting the editorship to some cis-atlantic physiologist, the American Publishers adopted the wiser plan of "making it worth the author's while to prepare a new edition specially for their press."

This acknowledgment of the "consideration" of the American Publishers, it is hoped, will evince a sufficient desire to remunerate English Authors for their labors, and remove at least a part of the accusations of "piracy," "theft," "huckstering other men's brains," &c., that have been so freely lavished upon us by those who felt themselves aggrieved in this matter.

By this arrangement the Author has been enabled to lay before the American reader the latest views both of himself and his fellow laborers in this most fascinating field of inquiry; it has also afforded him the opportunity of revising and modifying what had already been presented to them in previous editions.

The principal changes in the present edition are under the head of the Nervous System and in the chapter on Generation. (The latter title substituted for that of Reproduction.) It will be seen by reference to the first, that the Author has abandoned certain parts of Dr. M. Hall's doctrines, and substituted a "far simpler and more consistent view of the Constitution of the cerebro-spinal centres, which may be said to be based on the doctrines of Messrs. Todd and Bowman, though differing from them in some important particulars."

In the chapter on Generation the Author has substituted the views of Bischoff respecting the development of the ovum, for those of Dr. Barry's "having satisfied himself that the latter are no longer tenable." To Bischoff, he awards all the credit that he formerly gave to Barry. Those who are familiar with the previous editions will be struck with this change, and we think *favorably* impressed.

In glancing through the volume, one cannot fail to be struck with the evidences of great industry that meet him on every hand; nothing that has the least bearing on the subject seems to have escaped the author; all the latest discoveries and observations are scanned, and adopted or rejected as worthy or unworthy of credit; and the wonder is where a man of Dr. C.'s, numerous engagements can find the time to keep himself so fully "posted up."

It will be seen also that many of the old wood cuts have been omitted and those of a better character substituted, whilst the first lithographic plate has been replaced by another of "more accurate character and superior execution," all greatly enhancing the value of the work, and we rise from our hasty examination of the volume before us with the conviction that it presents the clearest and most comprehensive view of the existing state of Physiological Science of any work with which we are familiar.

